# A univariate and network analysis approach to studying connected speech in Subjective Cognitive Decline

**DOI:** 10.1002/alz70857_103284

**Published:** 2025-12-25

**Authors:** Sophie Pellerin, Simona Maria Brambati

**Affiliations:** ^1^ University of Montreal, Montréal, QC, Canada; ^2^ CRIUGM, Montréal, QC, Canada; ^3^ Université de Montréal, Montréal, QC, Canada; ^4^ Centre de Recherche de l'Institut Universitaire de Gériatrie de Montréal, Montréal, QC, Canada

## Abstract

**Background:**

Widespread Connected Speech (CS) changes (e.g., slower speech rate, more word repetitions, lower lexical diversity, reduced syntactic complexity) have been documented in Mild Cognitive Impairment (MCI). Nevertheless, the CS profile of individuals with Subjective Cognitive Decline (SCD; who are often considered to be at an even earlier stage of Alzheimer's disease (AD)), potential relationships between CS features in SCD, and how CS samples produced by individuals with SCD compare to those produced by controls and individuals with MCI remain unclear. The aim of this study was to compare the CS features and relationships between these features in SCD to those of controls and individuals with MCI.

**Method:**

Thirty CS features, part of all CS domains (e.g., fluency (e.g., filled pauses), lexical (e.g., word frequency), syntactic (e.g., subordinate clauses)) were extracted using Natural Language Processing techniques from the CS samples of 156 controls, 109 individuals with SCD, and 239 individuals with MCI. Groups were compared using ANCOVA models on the extracted CS features. Gaussian Graphical Models were then used to construct a CS network for each group with the CS features.

**Result:**

The ANCOVA analyses showed an increased speech rate (versus controls and individuals with MCI) and a lower local coherence (versus controls) in SCD. Moreover, our network analysis revealed increased (e.g., proportion of nouns, semantic idea density), decreased (e.g., proportions of pronouns and verbs), or intermediate (e.g., word valence) standardized node strength centralities in the SCD network compared to the Control and MCI networks. Examination of prominent edges in the SCD network revealed a similar pattern, with some increased (e.g., word frequency – noun valence), decreased (e.g., number of words – efficiency), and intermediate weights (e.g., word frequency – noun frequency) compared to the other networks.

**Conclusion:**

Our results suggest subtle CS changes in SCD, mainly in the lexical and semantic domains. Furthermore, our network analysis demonstrates that SCD represents an intermediate stage between healthy aging and MCI. Finally, we show that network analysis helps gain a different and more in‐depth understanding of CS in the early stages of the AD continuum.

